# A systematic review on mobile health applications for foodborne disease outbreak management

**DOI:** 10.1186/s12889-021-12283-6

**Published:** 2021-12-08

**Authors:** Genevie Ntshoe, Andronica Moipone Shonhiwa, Nevashan Govender, Nicola Page

**Affiliations:** 1 Division of Public Health Surveillance and Response, National Institute for Communicable Diseases, a division of the National Health Laboratory Service, Johannesburg, South Africa; 2 School of Health Systems and Public Health, Faculty of Health Sciences, University of Pretoria, Pretoria, South Africa; 3 Centre for Enteric Diseases, National Institute for Communicable Diseases, a division of the National Health Laboratory Service, Johannesburg, South Africa; 4 Department of Medical Virology, Faculty of Health Sciences, University of Pretoria, Pretoria, South Africa

**Keywords:** Foodborne diseases, Food poisoning, mHealth application, mobile application, Outbreak response system

## Abstract

**Background:**

Foodborne disease outbreaks are common and notifiable in South Africa; however, they are rarely reported and poorly investigated. Surveillance data from the notification system is suboptimal and limited, and does not provide adequate information to guide public health action and inform policy. We performed a systematic review of published literature to identify mobile application-based outbreak response systems for managing foodborne disease outbreaks and to determine the elements that the system requires to generate foodborne disease data needed for public action.

**Methods:**

Studies were identified through literature searches using online databases on PubMed/Medline, CINAHL, Academic Search Complete, Greenfile, Library, Information Science & Technology. Search was limited to studies published in English during the period January 1990 to November 2020. Search strategy included various terms in varying combinations with Boolean phrases “OR” and “AND”. Data were collected following the Preferred Reporting Items for Systematic Reviews and Meta-Analyses Statement. A standardised data collection tool was used to extract and summarise information from identified studies. We assessed qualities of mobile applications by looking at the operating system, system type, basic features and functionalities they offer for foodborne disease outbreak management.

**Results:**

Five hundred and twenty-eight (528) publications were identified, of which 48 were duplicates. Of the remaining 480 studies, 2.9% (14/480) were assessed for eligibility. Only one of the 14 studies met the inclusion criteria and reported on one mobile health application named MyMAFI (My Mobile Apps for Field Investigation). There was lack of detailed information on the application characteristics. However, based on minimal information available, MyMAFI demonstrated the ability to generate line lists, reports and offered functionalities for outbreak verification and epidemiological investigation. Availability of other key components such as environmental and laboratory investigations were unknown.

**Conclusions:**

There is limited use of mobile applications on management of foodborne disease outbreaks. Efforts should be made to set up systems and develop applications that can improve data collection and quality of foodborne disease outbreak investigations.

**Supplementary Information:**

The online version contains supplementary material available at 10.1186/s12889-021-12283-6.

## Background

Foodborne diseases are a growing public health concern globally with the ability to cause large scale outbreaks [1]. About 600 million foodborne diseases (FBDs) including over 400 000 deaths are reported worldwide [1]. In the World Health Organization (WHO) African region more than 91 million people are affected annually, 137 000 of whom die as a result of FBDs [1]. Nevertheless, there appears to be significant underreporting of foodborne disease (FBD) outbreaks in many parts of the world including South Africa. Many people do not seek medical care, as a result cases and outbreaks of FBD remain undetected.

FBDs are classified as one of the priority conditions in the African region including South Africa [2, 3]. In South Africa, a FBD outbreak is a category 1 Notifiable Medical Condition (NMC); as such healthcare professionals are required by law to immediately notify all cases by telephone after diagnosis followed by electronic notification within 24 hours of detection to relevant stakeholders [4]. FBD outbreaks are reported through the NMC Surveillance System (NMCSS) [5]. The NMCSS has been reengineered to improve reporting of notifiable medical conditions [6]. Prior to reengineering of the NMCSS, the system was paper-based; information required moved slowly, was incomplete and sometimes did not reach the intended individuals. The new NMCSS has two platforms: the paper-based and electronic system and notifications can be made through any of the platforms [7]. Even though FBD outbreaks are notifiable, the available surveillance data from the notification system is suboptimal and limited [6]. Focus of the NMCSS is currently on detecting and reporting cases, not on case investigation and response activities conducted during an outbreak [6]. As a result, FBD outbreaks that are reported are poorly investigated if they are investigated at all [8, 9].

There is an urgent need to identify rapidly and easily implementable strategies, and to develop systems that can detect, verify, respond and manage disease outbreaks in real time. Identifying these strategies are crucial in preventing widespread epidemics. Several studies have shown that it is possible to set up systems that can assist in coordinating outbreak response activities and resources needed for public health responses and data management using mobile health (mHealth) applications [10–13]. mHealth uses mobile devices such as mobile phones to gather and disseminate health-related information [14, 15]. There has been substantial investment in mHealth research globally. However, research in this field has been limited in low- and middle income countries. Nonetheless, several countries have successfully designed, piloted, evaluated and implemented use of mHealth applications for reporting, data collection, disease surveillance and outbreak management [10, 12, 16–19].

In South Africa, several mHealth applications are already in use, including those used in community based healthcare services and maternal and child health programmes [20]. However, there is limited use of mHealth application in disease outbreak management. We performed a systematic review of published literature to identify studies reporting on mobile applications for managing FBD outbreaks and mHeath tools used. In addition, we assessed the features and functionalities of the mobile applications to identify the attributes required to generate meaningful FBD data for public health action.

## Methods

### Search strategy and selection criteria

The search to identify studies was conducted by the two investigators (GN and AMS) in September and November 2020, respectively and date last searched was 27 November 2020. Studies were identified through literature searches using online databases from PubMed/Medline, CINAHL, Academic Search Complete, Greenfile, Library, Information Science & Technology. The following terms were included in the search and used in varying combinations with Boolean phrases “OR” and “AND”: mobile application OR web application OR web platform OR mobile health OR mHealth OR electronic health OR eHealth OR mobile device OR mobile phone OR cell phone OR cellular phone OR smart phone OR tablet AND outbreak investigation OR outbreak management OR outbreak response OR surveillance AND foodborne disease OR foodborne illness OR foodborne infection OR food poisoning. The search strategy used in MEDLINE via PubMed is shown in supplemental file 1.

The search was limited to studies published in English during the period January 1990 to November 2020. Two investigators (GN and AMS) independently screened titles and abstracts, assessed full text articles of eligible studies and selected studies that met the inclusion criteria. Where there was no consensus, both investigators had discussions with the senior author (NP) to review the findings and agreed on a joint assessment.

### Inclusion and exclusion criteria

Peer-reviewed publications reporting on mobile phone-based applications for investigation of foodborne disease outbreaks were included in the study. Studies published before the year 1990, those published in other languages, mobile applications not reporting on FBD outbreak management and FBD mHealth interventions that were not mobile-phone based applications (e.g. web-based applications) and those not focusing on outbreak investigation were excluded.

### Data extraction and assessment of functionalities

Data were collected following the Preferred Reporting Items for Systematic Reviews and Meta-Analyses (PRISMA) Statement [21]. Duplicate records were excluded. A standardised data collection tool was used to extract and summarise information from the studies identified. Two investigators (GN and NG) extracted data and assessed functionalities of the mobile applications identified. Data extracted and assessed included study characteristics i.e. study aim/purpose, study design and duration/period, country, participants, disease focus, type of intervention, mHealth tool/application name and data collection tool/type of mobile device used.

We also looked at the features of the mobile applications identified and assessed their qualities and key functionalities. *Characteristics* of the application included the following: (1) Operating system (Android / iPhone or Windows based platform), (2) System type (open or closed source platform, (3) Server characteristics (function as a cloud or client-based network), (4) App size and (5) Free subscription.

*Qualities* assessed included availability of the following functions: (1) security features, (2) reliability (work offline), (3) flexibility (adapt as technology evolve), (4) export data (generate line lists), (5) generate reports (basic descriptive stats) and (6) Two-way communication.

*Key functionalities* included availability of the following components: (1) Outbreak verification (verifying existence of an outbreak), (2) Epidemiological investigation (collection of information on demographic, clinical, exposure history and/or risk factor information and specimen details, (3) Environmental investigation (assessment of the environment and sampling), (4) Laboratory investigation (i.e. Information on testing of foodborne pathogens), (5) Laboratory data management (interface with laboratory information system to enable direct dispatching of results), (6) Decision support framework (ability to guide public health officials in decision making and tracking tasks).

Findings were reported following the PRISMA statement [21]. Study and application characteristics were indicated while qualities and key functionalities were assessed by a yes (feature available), no (feature not available) or unknown (absence of information/not clear whether the application encompasses those features or not) answer.

## Results

### Study selection

**Fig. 1:**
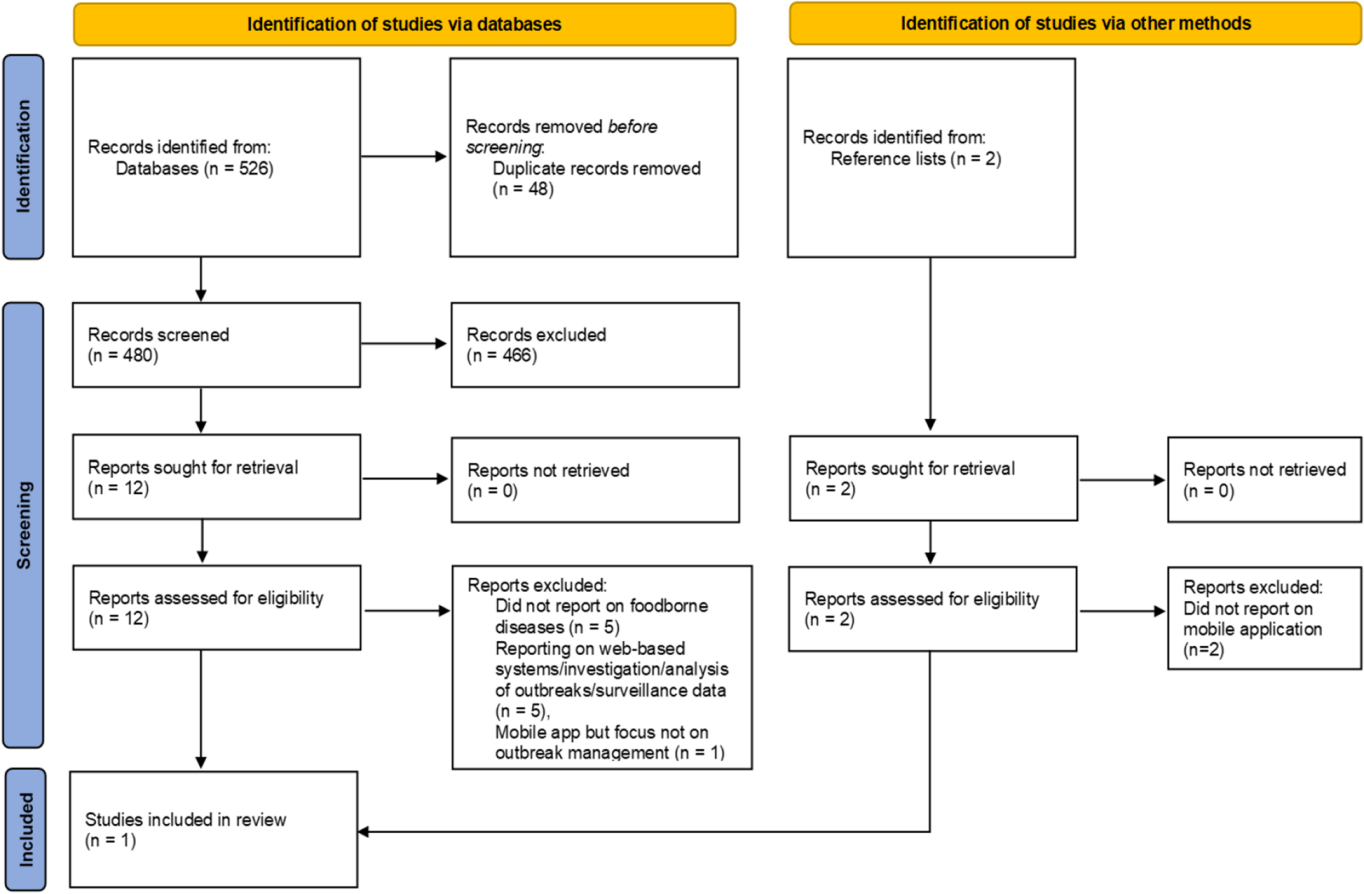
A flow diagram illustrating selection of publications on mHealth applications for foodborne disease outbreak management

A total of 528 publications was identified, of which 48 were duplicates (Fig. 1). Of the 480 remaining publications, 14 (2.9%) were assessed for eligibility while 466 were excluded based on relevance. Of the 14 publications assessed, one met the inclusion criteria and reported on a specific mHealth tool. Thirteen were excluded as focus was not on FBDs or FBD outbreak investigation. Where focus was on FBDs, either mobile application was not reporting on FBD outbreak investigation or identified systems were web-based not mobile phone-based (Fig. 1).

### Study characteristics

The study identified [22] was published in 2019 and aimed to evaluate the effectiveness of a mobile application named MyMAFI (My Mobile Apps for Field Investigation) in investigating FBD outbreaks. The study was a randomised cross-over trial with study duration of three months, conducted in Malaysia and participants were public health inspectors. Smartphones (Android phones) were used for data collection (Table 1). Training was conducted with participants prior to evaluating the application. However, assessment was conducted using FBD outbreak simulations and not an actual event of a FBD outbreak investigation.

**Table 1:** Characteristics of studies reporting on mobile applications for foodborne disease outbreak management/investigation

Study, Year, Country	Aim/purpose	Study design	Study period	Participants	Type of intervention, App name, App size	Mobile devices used
Hamzah et al. [22], 2019, Malaysia (Kelantan)	Assess effectiveness of a mobile-based application in data collection compared to paper-based format	Randomised Cross-over Trial	Sep-Nov 2018	Public health inspectors	Mobile application, MyMAFI (My Mobile Apps for Field Investigation), 3.3 megabytes	Smartphones/ Android phones

### Characteristics, capabilities and functionalities of MyMAFI

MyMAFI has an application size of 3.3 Megabytes, uses Android operating system and is available in Malay. System type and server characteristics were not indicated. Where information was available, MyMAFI demonstrated two capabilities and two key functionalities respectively. Capabilities included the ability to generate line lists, perform basic descriptive analyses and generate reports. Key functionalities specified included outbreak verification and epidemiological investigation. There was absence of information on security features, reliability, flexibility, feedback loops and components relating to environmental and laboratory investigations. Features on laboratory data management and decision support framework were not available (Table 2).

**Table 2:** Characteristics and key functionalities of mobile applications for foodborne disease outbreak management

Features	mHealth intervention/App name
	**MyMAFI**
Characteristics	
Free Subscription	Unknown
Open source platform	Unknown
Server characteristics	Unknown
Security features	Unknown
Reliability	Unknown
Flexibility	Unknown
Two-way communication	Unknown
Export data	Yes
Generate report	Yes
Key functionalities	
Outbreak verification	Yes
Epidemiological investigation	Yes
Environmental investigation	Unknown
Laboratory investigation	Unknown
Laboratory data management	No
Decision support	No

Yes: Feature available; No: Feature not available; Unknown: absence of information / not clear whether the application encompasses those features or not

## Discussion

To our knowledge, this article is the first to review mHealth applications for managing FBD outbreaks. We described characteristics of studies identified and assessed the features, capabilities and key functionalities of mobile applications currently available for FBD outbreak management. Only one study by Hamzah et al. met the inclusion criteria and reported on one specific mHealth application named MyMAFI that was developed to collect data during field investigation of FBD outbreaks in Malaysia [22]. The study [22] assessed the effectiveness of the mobile application in collecting data and MyMAFI was found to have improved timeliness of reporting as compared to the paper-based format. Findings by Hamzah et al. are similar to what has been reported in other studies reporting on the use of mobile applications for disease surveillance and outbreak response activities [23, 24].

MyMAFI operate on an Android platform and is available in one language Malay. Availability of the mobile application in one platform may lead to limited use of the application as public health officials utilising smartphones with iPhone operating system (iOs) will not be in a position to collect data during field investigation. As such, there is a need to encompass other operating systems and platforms such as iOs and web-based applications. Inclusion of other platforms would result in more coverage and lead to increased reporting and notifications as more people would have access to the application. There was absence of information on system type and server characteristics of MyMAFI. However, based on minimal information available, MyMAFI may have been developed on a closed source platform and function as a client-based network. mHealth interventions that are already in use for other disease outbreak management were developed on an open source platform [25–27]. Open source networks encourage collaboration as others can learn from and improve features and functionalities of respective applications that have been developed making them more sustainable.

There was limited information describing detailed qualities and key functionalities of MyMAFI. As a result, it was not possible to assess all attributes that MyMAFI offers. However, based on available information, basic elements constituting the standard investigation form for FBD with additional features were used to develop an application for field investigation. The basic elements in the investigation form usually include personal and clinical details, food history/risk factor information and in some specimen details for laboratory investigations. Features that were also added included automatic generation of line lists, epidemiological curve and descriptive analysis. Outbreak verification and epidemiological investigation were the key functionalities that were specified. There was no mention of other crucial components such as environmental and laboratory investigations. It is not clear whether these elements are available or not. Lack of these components would hamper identification of implicated food and pathogens that might be responsible for an outbreak.

An effective FBD outbreak system should incorporate key attributes on outbreak verification, epidemiological, environmental and laboratory investigations. These attributes are crucial in verifying existence, determining the possible source and cause of an outbreak, so that corrective measures can be implemented and prevent further spread. In addition, laboratory-based alerts are of importance as they facilitate immediate relaying of results so that appropriate case management and intervention measures can be initiated timeously to prevent further spread. Furthermore, provision of decision support framework will guide outbreak response teams in gathering information needed for public health action. Although laboratory data management and decision support framework may not be key attributes of a FBD outbreak response system, they are important key functionalities for disease outbreak management as they provide much needed info relating to pathogen/s causing disease and guidance to public health official in real time.

Even though only one mobile application for FBD outbreak investigation was identified, there have been advancements in recognising that mobile applications can be useful tools in collecting data related to foodborne diseases. In 2019, Seitzinger et al. published an article on a mobile application named Ethica that was assessed for data collection on food consumed and foodborne diseases but focus was not on FBD outbreak management [28]. The Ethica application was described to have improved data quality and displayed basic features such as reliability, flexibility, security features, feedback loops, generate reports and automatic updates. Additional features include ability to capture detailed food history in real time, store data for weeks when access to data is not available and availability of user and time-triggered interfaces to report illness. The Ethica application can operate on Android, iPhone and web-based platforms. Basic features of the Ethica application are in par with other outbreak management systems in use [27, 29]. The Ethica application has the potential to be used for FBD outbreak investigation if key attributes for FBD outbreak management can be incorporated on the application.

Furthermore, though limited, there are web-based platforms that have been in use for years for FBD surveillance and outbreak reporting in the United States [30, 31]. These platforms i.e. the National Outbreak Reporting System (NORS) and Foodborne Disease Outbreak Surveillance System (FDOSS) by the Centers for Disease Control and Prevention have proved to collect data needed for public health action. However, there is still limited use of web-based systems for FBD outbreak management in many parts of the world including South Africa. Platforms such as NORS/FDOSS can guide further development of other web-based systems or mobile applications in terms of features and components required to generate meaningful data during FBD outbreak investigations. Mobile applications are also of importance as they can be used to collect data in real time when officials are in the field. As FBD outbreaks are notifiable and common, it is important to have systems that will encompass both FBD surveillance and outbreak investigation as surveillance will lead to early detection and timely response to outbreaks.

Our study has several limitations. Firstly, our search was limited to scientific literature databases and did not include Google Scholar. As such, publications on this platform may have been missed. Secondly, inclusion criteria was limited to mobile phone-based applications for FBD outbreak, therefore it is not representative of all systems in use for collection of FBD data including surveillance. Web-based systems were excluded and this may have resulted in this review missing other features that may be useful for FBD outbreak investigation. Thirdly, only studies published in English were included and did not account for articles published in other languages. Fourthly, only one mobile-based application was identified and there was lack of published information regarding its detailed features and functionalities. As such, it was not possible to compare its characteristics with other applications making it difficult to determine if the application is in par with other mHealth application in use for disease outbreak management. Lastly, attempts were made through email and by telephone, multiple times, to contact the corresponding author of the MyMAFI paper to obtain more information; however response was not forthcoming. We also searched online and through app stores for the application to be able to reach out to the developers, but the application was not available through these searches.

## Conclusions

This review highlighted that there is still limited use of mobile applications for the management of FBD outbreaks worldwide. The review identified only one mobile application (MyMAFI), which demonstrated that use of mHealth applications can improve timeliness of reporting. However, additional improvements such as development of the application to suit other operating systems such as iOs, inclusion of other languages and other key attributes for FBD outbreak investigation and functionalities for outbreak management should be incorporated. Efforts should be made to set up systems and develop applications that can improve data collection and quality of foodborne disease outbreak investigations globally.

## Supplementary Information

**Additional file 1.**

**Additional file 2.**

## Data Availability

All information generated is included in this published article.

## Abbreviations

FBD: Foodborne disease
FDOSS: Foodborne Disease Outbreak Surveillance System
iOs: iPhone Operating System
mHealth: mobile health
MyMAFI: My Mobile Apps for Field Investigation
NORS: National Outbreak Reporting System
PRISMA: Preferred Reporting Items for Systematic Reviews and Meta-Analyses
