# Supplementary material for: A systematic review on mobile health applications for foodborne disease outbreak management
Source: BMC Public Health. 2021 Dec 8;21:2228. doi: 10.1186/s12889-021-12283-6 (PMC8653522; doi:10.1186/s12889-021-12283-6)
Supplement: Supplementary file 1 — Additional file 1. [file 12889_2021_12283_MOESM1_ESM.pdf]

Search strategy_MEDLINE via PubMed

Search Title/Abstract using the following terms with Boolean phrases "OR" and "AND"

1. mobile application OR web application OR web platform OR mobile health OR mHealth OR electronic health OR eHealth OR mobile device OR mobile phone OR cell phone OR cellular phone OR smart phone OR tablet

AND

2. outbreak investigation OR outbreak management OR outbreak response OR surveillance

AND

3. foodborne disease OR foodborne illness OR foodborne infection OR food poisoning

4. 1 AND 2 AND 3

Query

(((mobile application[Abstract] OR web application[Abstract] OR web platform[Abstract] OR mobile health[Abstract] OR mHealth[Abstract] OR electronic health[Abstract] OR eHealth[Abstract] OR mobile device[Abstract] OR mobile phone[Abstract] OR cell phone[Abstract] OR cellular phone[Abstract] OR smart phone[Abstract] OR tablet[Abstract])) AND (outbreak investigation[Abstract] OR outbreak management[Abstract] OR outbreak response[Abstract] OR surveillance[Abstract])) AND (foodborne disease OR foodborne illness OR foodborne infection OR food poisoning)

Search details

("mobile applications"[MeSH Terms] OR ("mobile"[All Fields] AND "applications"[All Fields]) OR "mobile applications"[All Fields] OR ("mobile"[All Fields] AND "application"[All Fields]) OR "mobile application"[All Fields] OR ("web"[All Fields] AND ("applicabilities"[All Fields] OR "applicability"[All Fields] OR "application"[All Fields] OR "applications"[All Fields] OR "applicative"[All Fields])) OR ("web"[All Fields] AND ("platform"[All Fields] OR "platform s"[All Fields] OR "platforms"[All Fields])) OR ("telemedicine"[MeSH Terms] OR "telemedicine"[All Fields] OR ("mobile"[All Fields] AND "health"[All Fields]) OR "mobile health"[All Fields]) OR ("mhealth s"[All Fields] OR "telemedicine"[MeSH Terms] OR "telemedicine"[All Fields] OR "mhealth"[All Fields]) OR (("electronical"[All Fields] OR "electronically"[All Fields] OR "electronics"[MeSH Terms] OR "electronics"[All Fields] OR "electronic"[All Fields]) AND ("health"[MeSH Terms] OR "health"[All Fields] OR "health s"[All Fields] OR "healthful"[All Fields] OR "healthfulness"[All Fields] OR "healths"[All Fields])) OR ("telemedicine"[MeSH Terms] OR "telemedicine"[All Fields] OR "ehealth"[All Fields]) OR (("mobile"[All Fields] OR "mobiles"[All Fields]) AND ("device s"[All Fields] OR "equipment and supplies"[MeSH Terms] OR ("equipment"[All Fields] AND "supplies"[All Fields]) OR "equipment and supplies"[All Fields] OR "device"[All Fields] OR "instrumentation"[MeSH Subheading] OR "instrumentation"[All Fields] OR "devices"[All Fields])) OR ("cell phone"[MeSH Terms] OR ("cell"[All Fields] AND "phone"[All Fields]) OR "cell phone"[All Fields] OR ("mobile"[All Fields] AND "phone"[All Fields]) OR "mobile phone"[All Fields]) OR ("cell phone"[MeSH Terms] OR ("cell"[All Fields] AND "phone"[All Fields]) OR "cell phone"[All Fields]) OR ("cell phone"[MeSH Terms] OR ("cell"[All Fields] AND "phone"[All Fields]) OR "cell phone"[All Fields] OR ("cellular"[All Fields] AND "phone"[All Fields]) OR "cellular phone"[All Fields]) OR ("smartphone"[MeSH Terms] OR "smartphone"[All Fields] OR ("smart"[All Fields] AND "phone"[All Fields]) OR "smart phone"[All Fields]) OR ("tablet s"[All Fields] OR

"tabletability"[All Fields] OR "tableted"[All Fields] OR "tablets"[MeSH Terms] OR "tablets"[All Fields] OR "tablet"[All Fields] OR "tableting"[All Fields] OR "tabletted"[All Fields] OR "tabletting"[All Fields])) AND ((("disease outbreaks"[MeSH Terms] OR ("disease"[All Fields] AND "outbreaks"[All Fields]) OR "disease outbreaks"[All Fields] OR "outbreak"[All Fields] OR "epidemiology"[MeSH Subheading] OR "epidemiology"[All Fields] OR "outbreaks"[All Fields] OR "outbreak s"[All Fields]) AND ("investigated"[All Fields] OR "investigates"[All Fields] OR "investigating"[All Fields] OR "investigation"[All Fields] OR "investigations"[All Fields] OR "investigative"[All Fields] OR "investigator s"[All Fields] OR "research personnel"[MeSH Terms] OR ("research"[All Fields] AND "personnel"[All Fields]) OR "research personnel"[All Fields] OR "investigator"[All Fields] OR "investigators"[All Fields])) OR (("disease outbreaks"[MeSH Terms] OR ("disease"[All Fields] AND "outbreaks"[All Fields]) OR "disease outbreaks"[All Fields] OR "outbreak"[All Fields] OR "epidemiology"[MeSH Subheading] OR "epidemiology"[All Fields] OR "outbreaks"[All Fields] OR "outbreak s"[All Fields]) AND ("manage"[All Fields] OR "managed"[All Fields] OR "management s"[All Fields] OR "managements"[All Fields] OR "manager"[All Fields] OR "manager s"[All Fields] OR "managers"[All Fields] OR "manages"[All Fields] OR "managing"[All Fields] OR "managment"[All Fields] OR "organization and administration"[MeSH Terms] OR ("organization"[All Fields] AND "administration"[All Fields]) OR "organization and administration"[All Fields] OR "management"[All Fields] OR "disease management"[MeSH Terms] OR ("disease"[All Fields] AND "management"[All Fields]) OR "disease management"[All Fields])) OR (("disease outbreaks"[MeSH Terms] OR ("disease"[All Fields] AND "outbreaks"[All Fields]) OR "disease outbreaks"[All Fields] OR "outbreak"[All Fields] OR "epidemiology"[MeSH Subheading] OR "epidemiology"[All Fields] OR "outbreaks"[All Fields] OR "outbreak s"[All Fields]) AND ("response"[All Fields] OR "responses"[All Fields] OR "responsive"[All Fields] OR "responsiveness"[All Fields] OR "responsivenesses"[All Fields] OR "responsives"[All Fields] OR "responsivities"[All Fields] OR "responsivity"[All Fields])) OR ("epidemiology"[MeSH Subheading] OR "epidemiology"[All Fields] OR "surveillance"[All Fields] OR "epidemiology"[MeSH Terms] OR "surveilance"[All Fields] OR "surveillances"[All Fields] OR "surveilled"[All Fields] OR "surveillence"[All Fields])) AND ("foodborne diseases"[MeSH Terms] OR ("foodborne"[All Fields] AND "diseases"[All Fields]) OR "foodborne diseases"[All Fields] OR ("foodborne"[All Fields] AND "disease"[All Fields]) OR "foodborne disease"[All Fields] OR ("foodborne diseases"[MeSH Terms] OR ("foodborne"[All Fields] AND "diseases"[All Fields]) OR "foodborne diseases"[All Fields] OR ("foodborne"[All Fields] AND "illness"[All Fields]) OR "foodborne illness"[All Fields]) OR (("foodborn"[All Fields] OR "foodborne"[All Fields]) AND ("infect"[All Fields] OR "infectability"[All Fields] OR "infectable"[All Fields] OR "infectant"[All Fields] OR "infectants"[All Fields] OR "infected"[All Fields] OR "infecteds"[All Fields] OR "infectibility"[All Fields] OR "infectible"[All Fields] OR "infecting"[All Fields] OR "infection s"[All Fields] OR "infections"[MeSH Terms] OR "infections"[All Fields] OR "infection"[All Fields] OR "infective"[All Fields] OR "infectiveness"[All Fields] OR "infectives"[All Fields] OR "infectivities"[All Fields] OR "infects"[All Fields] OR "pathogenicity"[MeSH Subheading] OR "pathogenicity"[All Fields] OR "infectivity"[All Fields])) OR ("foodborne diseases"[MeSH Terms] OR ("foodborne"[All Fields] AND "diseases"[All Fields]) OR "foodborne diseases"[All Fields] OR ("food"[All Fields] AND "poisoning"[All Fields]) OR "food poisoning"[All Fields]))

Filters

Comment, Congress, Editorial, Journal Article, Systematic Review, Humans, English, MEDLINE, from 1990 - 2020
