# Supplementary material for: A systematic review on mobile health applications for foodborne disease outbreak management
Source: BMC Public Health. 2021 Dec 8;21:2228. doi: 10.1186/s12889-021-12283-6 (PMC8653522; doi:10.1186/s12889-021-12283-6)
Supplement: Supplementary file 2 — Additional file 2. [file 12889_2021_12283_MOESM2_ESM.pdf]

Supplemental file 2_Full text assessed and excluded

| Title | Authors | Citation | Journal | Publication Year |
|---|---|---|---|---|
| A Cross-Cutting Approach to Surveillance and Laboratory Capacity as a Platform to Improve Health Security in Uganda | Lamorde M, Mpimbaza A, Walwema R, Kamya M, Kapisi J, Kajumbula H, Sserwanga A, Namuganga JF, Kusemererwa A, Tasimwa H, Makumbi I, Kayiwa J, Lutwama J, Behumbiize P, Tagoola A, Nanteza JF, Aniku G, Workneh M, Manabe Y, Borchert JN, Brown V, Appiah GD, Mintz ED, Homsy J, Odongo GS, Ransom RL, Freeman MM, Stoddard RA, Galloway R, Mikoleit M, Kato C, Rosenberg R, Mossel EC, Mead PS, Kugeler KJ. | Health Secur. 2018 Fall;16(S1):S76-S86. doi: 10.1089/hs.2018.0051. | Health Secur | 2018 |
| Analyses of the eFORS (Electronic Foodborne Outbreak Reporting System) surveillance data (2000-2004) in school settings | Venuto M, Halbrook B, Hinners M, Lange A, Mickelson S. | J Environ Health. 2010 Mar;72(7):8-13. | J Environ Health | 2010 |
| Assessing the Concepts and Designs of 58 Mobile Apps for the Management of the 2014-2015 West Africa Ebola Outbreak: Systematic Review. | Tom-Aba, Daniel; Nguku, Patrick Mboya; Arinze, Chinedu Chukwujekwu; Krause, Gerard | JMIR Public Health Surveill. 2018; 4(4): e68 | JMIR Public Health and Surveillance | 2018 |

| | | | | |
|---|---|---|---|---|
| Compliance Rates, Advantages, and Drawbacks of a Smartphone-Based Method of Collecting Food History and Foodborne Illness Data | Seitzinger P, Osgood N, Martin W, Tataryn J, Waldner C. | J Food Prot. 2019 May 24:1061-1070. doi: 10.4315/0362-028X.JFP-18-547. Online ahead of print. | J Food Prot | 2019 |
| Daily zero-reporting for suspect Ebola using short message service (SMS) in Guinea-Bissau. | Cáceres, V. M.; Cardoso, P.; Sidibe, S.; Lambert, S.; Lopez, A.; Pedalino, B.; Guibert, D. J. Herrera | Public Health. 2016 Sep;138:69-73. doi: 10.1016/j.puhe.2016.03.006. Epub 2016 Apr 19. | Public Health (Elsevier) | 2016 |
| GenomeGraphR: A user-friendly open-source web application for foodborne pathogen whole genome sequencing data integration, analysis, and visualization | Sanaa M, Pouillot R, Vega FG, Strain E, Van Doren JM. | PLoS One. 2019 Feb 28;14(2):e0213039. doi: 10.1371/journal.pone.0213039. eCollection 2019. | PLoS One | 2019 |
| Investigating an outbreak of Clostridium perfringens gastroenteritis in a school using smartphone technology, London, March 2013 | Simone B, Atchison C, Ruiz B, Greenop P, Dave J, Ready D, Maguire H, Walsh B, Anderson S. | Euro Surveill. 2014 May 15;19(19):20799. doi: 10.2807/1560-7917.es2014.19.19.20799. | Euro Surveill | 2014 |
| Symptom monitoring of childhood illnesses and referrals: A pilot study on the feasibility of a mobile phone-based system as a disease surveillance tool in a rural health district of Ghana. | Mohammed, Aliyu; Acheampong, Princess Ruhama; Otupiri, Easmon; Owusu-Dabo, Ellis | Health Informatic J. 2020 Jun;26(2):1465-1476. doi: 10.1177/1460458219879329.Epub 2019 Oct 23 | Health Informatics Journal | 2020 |

| | | | | |
|---|---|---|---|---|
| The Pathogen-annotated Tracking Resource Network (PATRN) system: a web-based resource to aid food safety, regulatory science, and investigations of foodborne pathogens and disease | Gopinath G, Hari K, Jain R, Mammel MK, Kothary MH, Franco AA, Grim CJ, Jarvis KG, Sathyamoorthy V, Hu L, Datta AR, Patel IR, Jackson SA, Gangiredla J, Kotewicz ML, LeClerc JE, Wekell M, McCardell BA, Solomotis MD, Tall BD. | Food Microbiol. 2013 Jun;34(2):303-18. doi: 10.1016/j.fm.2013.01.001. Epub 2013 Jan 23. | Food Microbiol | 2013 |
| Use of mobile phones in an emergency reporting system for infectious disease surveillance after the Sichuan earthquake in China. | Yang C; Yang J; Luo X; Gong P | Bulletin of the World Health Organisation 2009; 87:619-623. doi:10.2471/BLT.08.060905 | Bulletin of the World Health Organization | 2009 |
| Using the Electronic Foodborne Outbreak Reporting System (eFORS) to improve foodborne outbreak surveillance, investigations, and program evaluation | Middaugh JP, Hammond RM, Eisenstein L, Lazensky R. | J Environ Health. 2010 Sep;73(2):8-11. | J Environ Health | 2010 |
| Acute Gastroenteritis Surveillance through the National Outbreak Reporting System, United States | Hall AJ, Wikswo ME, Manikonda K, Roberts VA, Yoder JS, Gould LH | Emerging Infectious Diseases. 2013 Aug;19(8) | Emerging Infectious Diseases | 2013 |
| A Platform for Crowdsourced Foodborne Illness Surveillance: Description of Users and Reports | Quade P, Nsoesie E O | JMIR Public Health Surveill. 2017 Jul-Sep; 3(3): e42 | JMIR Public Health and Surveillance | 2017 |
